# The Tension between National and Local Concerns in Preparing for Large-Scale Generic Systems in Healthcare

**DOI:** 10.1007/s10606-022-09424-9

**Published:** 2022-03-16

**Authors:** Gunnar Ellingsen, Morten Hertzum, Line Melby

**Affiliations:** 1 UiT - The Arctic University of Norway, Tromsø, Norway; 2 University of Copenhagen, Copenhagen, Denmark; 3 SINTEF Digital, Trondheim, Norway

**Keywords:** Large-scale generic systems, Electronic health records, Epic, General practitioners, National concerns, Local concerns, Central Norway

## Abstract

Large-scale generic systems are typically adapted to local practice through configuration. This is especially important in healthcare, which involves a plurality of institutions and users. However, the decision to acquire a generic system in public healthcare is typically founded on regional and national health policy goals, which often are translated into various forms of standardization. As a result, national and regional health policy interests may stand in contrast to interests on the local level. Therefore, we analyze how national and local concerns are weighed against each other in the preparations for implementing large-scale generic systems in healthcare. We explore what role configuration plays and what the prospects are for long-term development. We contribute with insight into how the organizational consequences of generic systems are formed already in the preparation phase and point to how configuration easily results in standardization, thereby basically privileging national and regional health goals at the expense of local needs. Empirically, we focus on the preparations for implementing the Epic electronic health record in Central Norway.

## Introduction

The implementation of large generic systems in organizations is associated with many benefits. Some of these are institution-wide coverage, streamlined work practices, and the possibility to reuse systems across institutional settings. However, many studies have noted how organizations differ and may have diverging needs (e.g., Berg and Goorman 1999; Star and Ruhleder 1996). To handle this diversity, generic systems have extensive configuration facilities (Cooney et al. 2011). These facilities make it possible to adapt the systems to diverse practices and users (Pollock et al. 2003), thereby minimizing the need for new development. In this process, the customer’s implementation program collaborates with the vendor about adapting the system to the organization and its users. In the preparation phase of implementing generic systems, users are typically invited to participate extensively. This way, they specify local needs and learn about technical possibilities. On this basis, the system is configured.

Generic systems have increasingly made their way into the health market. Some notable examples are the US-based companies Epic, Cerner, and Allscripts, which now are key players in the European healthcare market (Healthcare IT News 2019). This is also reflected in recent Epic electronic health record (EHR) installations in the Nordic countries. All these systems have well-developed configuration facilities, and as such, they “promise” to meet the needs of diverse users.

While the decision to acquire a generic system is generally founded on strategic considerations for the organization and management (Haines 2009; Poba-Nzaou et al. 2014), acquiring a generic system in a public healthcare system is typically founded on regional and national health policy goals (Hertzum and Ellingsen 2019). This implies that implementation programs also must adhere to these goals when planning the implementation of the generic system. Often, the national and regional goals are translated into various forms of standardization, such as standardization of the EHR content, standardized patient pathways, and standardization of clinical work.

Not surprisingly, interest on the national level may stand in contrast to interests on the local level. As a result, implementation programs are in a precarious position. On the one hand, they need to accommodate many specific and different local user requirements. On the other hand, they must align with national and regional health policy goals (which often means standardization). Accordingly, preparing for and implementing a generic system entail an inherent tension between national and local perspectives. Balancing these perspectives is fundamental to the successful implementation of generic systems. Therefore, we ask the three following research questions: How are national and local concerns weighed against each other in the preparations for implementing large-scale generic systems? What role does configuration play in this process? And what are the prospects for long-term user-controlled evolution of the system?

We respond to these questions by analyzing expectations and planning in the preparation phase. This phase is critical because the implementation of generic systems requires considerable planning – say several years - which may lead the various stakeholders to reflect on the generic system’s aim and potential consequences. In turn, these reflections could be valuable input for adjusting the program. We contribute insight into how the organizational consequences of generic systems are formed in the preparation phase. Furthermore, we discuss how configuration easily results in standardization, thereby basically privileging national and regional health goals at the expense of local needs.

Empirically, we focus on the preparations for the implementation of the Epic EHR in Central Norway. Given Epic’s all-encompassing scope, its widespread use in Western healthcare, and its considerable configurability, it is a prime example of a generic system. We interviewed representatives of the Health Platform program, which is responsible for preparing and managing the implementation of Epic. The Health Platform program envisages seamless access to information, process standardization, and work reallocation. We also interviewed representatives of the general practitioners (GPs), who provide primary healthcare to citizens through clinics run by a single GP or small group of GPs. As private entrepreneurs, each GP clinic decides whether to adopt Epic, but the Health Platform program has the main say in how Epic is configured.

Conceptually, we draw on the CSCW field, which has a long tradition of attending to the users’ perspective in local practice (Fitzpatrick and Ellingsen 2013; Orlikowski 1992). To account for the size and scope of the Epic EHR, we also draw on the information infrastructure concept, which is very useful for analyzing large-scale information systems (Aanestad and Jensen 2011; Bossen and Markussen 2010; Hanseth and Lyytinen 2010; Star and Ruhleder 1996).

The paper is organized as follows: The next section describes the theoretical framework and concepts used in this paper. The method section describes and explains the methods used in the empirical study. The background and findings sections give the context of the empirical study and present the findings resulting from it. These sections are followed by the discussion and the conclusion.

## Theory

Large-scale generic systems come under many names, for instance, packaged software, information system packages, enterprise resource planning (ERP) systems, enterprise systems (ES), or suite systems. A common feature, however, is that such systems are considered the backbone of the digital infrastructure of sizeable organizations where they seamlessly integrate information and work processes within and across organizational areas (Kumar and van Hillegersberg 2000, p. 23). Bygstad (2017) denotes such systems as heavy-weight IT and describes them as fully integrated solutions that depend on specialized IT competences for their reliability and security. They are also referred to as standardized, off-the-shelf software packages that build on best practices (Osnes et al. 2018). Some examples are the German SAP (Shim and Shim 2018), the US-based Epic EHR system (Davis and Khansa 2016), and Oracle (Cooney et al. 2011).

A key capability of generic systems is their extensive configuration facilities (Cooney et al. 2011; Pollock et al. 2007). These facilities make it possible to adapt the systems to diverse practices and users (Pollock et al. 2003), thereby minimizing the need for new development. In this regard, Pries-Heje and Dittrich (2009) argue that the generic system is only half a product since a substantial part of the design (i.e. adaptation) is deferred to the implementation process, or even later as part of actual use (Germonprez et al. 2011; Torkilsheyggi and Hertzum 2017). This emphasizes that generic systems never come as ready-to-use systems (Germonprez et al. 2011; Haines 2009; Torkilsheyggi and Hertzum 2017); they are instead configurable to enable their adaptation to various domains and customers. Configuration is thus expected to minimize the need for new software development, keep the software standardized, and make customers less reliant on the vendor for (re-)configuration of the system (Pollock et al. 2003). The possibility for users to participate in configuration processes is considered a key factor in motivating user groups to adopt the system (Markus and Mao 2004).

Generic systems have increasingly become part of the health market. The international companies Epic, Cerner, and Allscripts are the largest players in the US healthcare market with a market share of 28, 26, and 9%, respectively (Business insider 2020). Recent estimates suggest that the US EHR market will grow by 41% with a leap of over $3 billion in revenue between 2019 and 2025 (Arizton Advisory and Intelligence 2020). The above-mentioned companies all have a global outreach. They are also increasingly making inroads into the European market in competition with other large-scale contenders such as Chipsoft, MediSys, Dedalus, and Philips (Healthcare IT News 2019). Epic has also found its way into the Nordic countries, with installations in Finland and Denmark and a planned implementation in Norway in 2022.

Unfortunately, several large-scale system initiatives in healthcare have been seriously troubled or even failed. For example, Sholler (2020) points to how $25 billion of federal funding between 2011 and 2014 to implement EHRs in the US resulted in organized resistance among healthcare professionals. In the United Kingdom, the National Health Service (NHS) invested staggering £12.4 billion on a ‘one-size-fits-all’ EHR system, which was eventually scrapped in 2010 (Gov.UK 2021). At its startup in 2002, this program represented the largest non-military procurement in the world (Pollock and Williams 2010). In Denmark, the EUR 375 million implementation of Epic faced serious trouble (Hertzum and Ellingsen 2019). Two years after its startup in 2016, productivity was still lagging behind baseline.

While this trend for large-scale generic systems in healthcare should be of interest to the CSCW field, there have so far been very few studies on this topic. Fitzpatrick and Ellingsen (2013, p. 635) point to that

‘[Studies in the CSCW field] have been very good at describing (...) the healthcare setting from a workpractice perspective, but they have been far less concerned about analyzing the (larger) change processes associated with policy formation and the subsequent acquisition and implementations of large-scale ICT systems.’

It seems that the current status of the CSCW field is similar, although a handful of studies at the national level have now been carried out: Grisot and Vassilakopoulou (2017) analyzed the development of national, public eHealth services in Norway, i.e. web-based capabilities for communication between citizens and primary healthcare practitioners. Ulriksen et al. (2017) studied a national initiative in Norway aiming at standardizing the EHR content on the basis of the openEHR framework. Finally, Sholler (2020) studied how the US government’s policy-driven EHR infrastructure program - aimed to support data collection and sharing within and among healthcare organizations - resulted in organized resistance from healthcare professionals.

However, none of these studies focuses on the actual acquisition of large-scale generic systems and their formative stages. This points to a gap in the CSCW literature: Key conditions for successful use are already established in the formative stages of large-scale projects where many crucial decisions are made (Monteiro et al. 2013). These decisions will shape users’ expectations of what the generic system will entail (Hertzum et al. 2021). In this connection, a core issue in the larger, politically imbued process is how new technology and associated new practices lead to disagreements, controversies, and negotiations (Bossen and Markussen 2010; Latour 1987). This is especially characteristic of the healthcare sector with its many powerful professionals, each with their special needs. Given the size and scope of generic systems, there are also often several autonomous health institutions involved, all of which must be convinced that the new system will provide benefits for them before they will participate. While a formative evaluation approach cannot avoid the issues associated with large-scale projects, it can help navigate the associated complexities by capturing the perceptions of the stakeholders involved and feeding findings back to program management (Cresswell et al. 2020, p. 2).

To get a better understanding of large-scale generic systems in healthcare, we need a theoretical concept that focuses on large-scale issues and has a far-reaching scope. In this regard, it is particularly useful to apply the concept of information infrastructure. Information infrastructure has been used to study the design, implementation, and use of large-scale information systems (Aanestad and Jensen 2011; Hanseth and Lyytinen 2010; Star and Ruhleder 1996).

Systems in an information infrastructure are never standalone entities; they are rather integrated with other information systems and deeply embedded in conventions and practices (Aanestad and Jensen 2011). In healthcare, this means that an information infrastructure consists of a range of systems, health professionals, institutions, and established practices. Implementing a generic system in such a context implies replacing important parts of the existing information infrastructure. To do so successfully, the generic system must be configured for the local practices. While this is a touted capability of generic systems, they are still packaged solutions that prescribe certain use patterns. In the preparation of their implementation, it is therefore difficult to foresee all the existing dependencies and consequences as well as plan for all eventualities.

The collaboration among user groups in an information infrastructure occurs through conventional practices and regulations and may be supported by various technological and organizational means such as local EHRs, phones, meetings etc. Generally, information infrastructure does not have a centralized governanace structure. This is also the case in our study. The municipalities are usually self-governed political units, GPs are individual entrepreneurs, and the regional health authorities govern the hospitals. The distributed governance of the information infrastructure may constitute a major challenge for a generic system that promises a seamless information flow. It is difficult to instruct relatively autonomous user groups to opt-in; they must rather be motivated to participate through attractive incentive schemes.

A basic principle of an information infrastructure is that it is never built from scratch; rather, it grows through evolution of the installed base (Aanestad et al. 2017). That is, the infrastructure is in an ongoing co-construction process through which it evolves its technical and social elements (Monteiro et al. 2013; Star and Ruhleder 1996). During this evolution, the installed base grows and increasingly influences its environment. As a result, the installed base is difficult to replace or change. Therefore, newer parts of an installed base are carefully introduced or adjusted in a stepwise manner (Bowker and Star 1999). This is a process of ongoing negotiation and compromise among various stakeholders to achieve stability and alignment (Latour 1987). This evolutionary process stands in contrast to plans for short implementation schedules where a generic system replaces important parts of the installed base in one operation. Such plans involve increased risk of failure and unexpected side effects (Hertzum and Ellingsen 2019).

When analyzing large-scale information infrastructures, Star and Ruhleder (1996, p. 114) remind us that

‘An infrastructure occurs when the tension between local and global is resolved. That is, an infrastructure occurs when local practices are afforded by a larger-scale technology, which can then be used in a natural, ready-to-hand fashion.’

In this regard, large-scale generic systems in healthcare are not simply acquired to fit the users’ present needs and tasks. These systems also have an important strategic dimension for national health authorities. This typically involves utilizing technology for streamlining healthcare services, standardizing work processes that cut across organizational domains (Saxena and McDonagh 2018), standardized patient pathways (Delilovic et al. 2019), and greater ﻿structuring of the EHR content for clinical decision-support (Morrison et al. 2013). The strategic dimension in our case is strongly linked to the national goal of “one citizen - one record” (The Norwegian Directorate of eHealth 2018). If successful, it is considered to be the first step in achieving this goal (Central Norway Regional Health Authority 2021).

National interests are typically manifested in thorough requirements specifications and preparations for the generic systems (Ellingsen and Hertzum 2020). This implies that detailed requirements specifications and time-consuming negotiations with potential vendors involve both national and local concerns (Ellingsen and Hertzum 2020). This way, frictions between national and local interests are laid bare already in the preparation phase. The customer’s implementation program must translate national goals into numerous decisions about the system functionality and setup and ensure that the users get functionality tailored for their local practices. The customer’s implementation program is typically caught in the middle and must carefully balance these two positions. There may be no stable way to reconcile these poles due to the shifting influences of stakeholder groups with conflicting interests (Cresswell et al. 2020, p. 2). The NHS-initiated EHR program mentioned above provides an illustration. Initially, there was a strong pull toward national priorities, with a strategy that focused on joint procurement and interoperability. In the later years of the program, attention changed toward more local priorities (ibid.).

Overall, there are many questions and challenges related to large-scale generic systems in healthcare, and the global/local gap may be hard to reconcile. In this regard, Coiera (2009) questions the feasibility for large, complicated top-down IT implementations due the scale and inertia of such systems, which will have a limited capacity to adapt quickly to the significant delivery challenges the health service will face in the next 20 years. Nonetheless, studies based on information infrastructure may be a way to increase our understanding of the challenges associated with the acquisition of generic systems.

## Method

Empirically, we focus on the Health Platform program in Central Norway and its preparations for the implementation of the US-based EHR suite Epic. The study is based on an interpretive research tradition (Klein and Myers 1999; Walsham 1995), where reality is socially constructed among the participants. Interpretive research approaches the understanding of social processes by getting involved inside the world of those generating them, rather than by hypotheses-driven deductions or predefined variables. Conforming to this approach, we aim to see the phenomenon from the perspectives of different informants or stakeholders while also taking into account the broader context (Klein and Myers 1999; Forsythe 1999). Data gathering in interpretive research is generally based on interviews, participant observations, document analyses, and informal discussions (Klein and Myers 1999).

Our data collection extends over the period 2018–2021 and is based on various information sources: interviews, public reports, media coverage, and national policy documents. As a result of the scope and ambition of the Health Platform, we found it useful to target top management first to gain a comprehensive understanding of the program.

In a broad sense, the initial focus was on potentials and challenges with Epic. Through the interviews with the management, it became increasingly clear that the management believed that a crucial prerequisite for the program’s success was the participation of the GPs, given their coordinating role in following up patients in the health sector. In connection with this, the management explained that the GPs’ existing EHR was quite good, and thus pointed out that it would require persuasion to ensure their commitment to the program. As a result, we found it interesting to target GPs in our next round of interviews.

In the spring of 2019, the third author interviewed nine GPs in the health region. We chose GPs with long and broad experience. The interviewees had an average of 9 years of experience as GPs and worked in GP clinics that had 3–6 GPs. All the interviewees had previous experience from specialist health services. The interview questions were about the GPs’ current work situations, experiences with their existing EHR, and expectations of Epic. The interviews lasted 25–40 min, were audio-recorded and transcribed in their entirety. Subsequently, the third author asked some follow-up questions to get further details about causes and relations.

The interviews with both the top managers and the GPs were conducted in an open-ended manner, which provided the interviewees with the opportunity to organize their answers within their own frameworks. The five managers are quoted as follows: The manager having the clinical responsibility for the specialist health service is referred to as “Director, medical hospitals”. The manager having the clinical responsibility for the GPs in the municipal health service is referred to as “Director, medical GP”. The manager having the responsibility for the home-care sector and nursing homes in the municipal health service is referred to as “Director, municipalities”. The manager having the responsibility for the benefits realization is referred to as “Director, benefits realization” and the top manager is referred to as “Director, top”. The quoted GPs are referred to as “GP-1”, “GP-2”, etc.

The following data serve as secondary sources for the article: In the autumn of 2020, the first author conducted ten interviews with health personnel and consultants in one municipality in North Norway. Although this was outside the Central Norway region in which Epic is being implemented, it was about the related topic of national ambitions for a common EHR for the entire Norwegian municipal health sector apart from Central Norway. In addition, the first author interviewed the representatives of the top management of the three principal vendors of EHR systems to the Norwegian municipal healthcare market. These interviews focused on the future market situation for these vendors in connection with the implementaton of Epic.

The analysis of the data is based on a hermeneutic approach and the principle of the hermeneutic circle. This means that we come to understand a complex whole from preconceptions about the meanings of its parts and their interrelations. Klein and Myers (1999, p. 71) consider this principle as the first (and most important) of seven principles for conducting interpretive field research. Related to our study, the Health Platform can be analyzed from both the top management’s perspective and the GPs’ perspective. Accordingly, to improve our understanding of the program as a whole, we shifted our gaze back and forth in an iterative way between viewpoints at the local and regional/national level.

The second principle of contextualization requires critical reflection on the research setting’s social and historical background. In the background section, we contextualize the Health Platform program and elaborate on the current situation for the GPs.

The third principle of interaction between the researchers and the study participants relates to the emergence of the four categories we apply in our case. The first two categories, “Accessing patient information anytime, anywhere” and “Standardizing patient pathways” are strongly associated with the goals of the project and were clearly expressed in the interviews with the top management. The third category, “An EHR that responds to evolving needs”, evolved out of the interviews with the GPs when they expressed what type of EHR they would like to have for the future and what they wouldn’t. The fourth category on “Relocating work” represented a future ambition for the top management but emerged as an imminent threat to the GPs. In the analytical process, we discussed the empirical data thoroughly among ourselves, and we strived for a balanced picture of what was happening. Right from transcribing the first interviews, we also looked for recurrent themes and patterns, which we explored further in the following interviews.

The fourth principle of abstraction and generalization connects to our use of the information infrastructure concept. We have elaborated on this choice in the theory section. But it is fair to say that the information infrastructure concept informed our analytical categories in the discussion, especially on strategies for dealing with large-scale systems in healthcare.

The fifth principle of dialogical reasoning “requires sensitivity to possible contradictions between the theoretical preconceptions guiding the research design and actual findings” (ibid., p. 72). In our first round of reviewing, the reviewers challenged our choice of theoretical framework; based on this input, we realized that the information infrastructure concept was a better fit.

The sixth principle of multiple interpretations is in our case accounted for by the difference in views between the top management and the GPs.

The seventh principle of suspicion requires sensitivity to possible “biases” and systematic “distortions” in the narratives collected from the participants. We did not find this to be an issue.

## Background

The Health Platform is a regional program owned by the Central Norway Regional Health Authority and Trondheim municipality. In 2019 it signed a contract with Epic Systems Corporations on acquiring and implementing the Epic EHR in the whole region, including all the hospitals, GP clinics, nursing homes, and home-care services. Given the scope and ambition of the program, the Health Platform is also a pilot for the national goal of “one citizen - one record” (The Norwegian Directorate of eHealth 2018). An arrangement with the Norwegian state ensures that the municipalities may finance the investment costs through a loan from the Norwegian Government (Hertzum and Ellingsen 2019, p. 315). However, recently the county council of Trøndelag (the largest county in Central Norway) encouraged the Norwegian Association of Local and Regional Authorities to engage themselves with the Government to ensure that a greater part of the costs are financed over the Norwegian state budget (Norwegian Association of Local and Regional Authorities 2021). In turn, this has resulted in a formal request to Norway’s new Minister of Health to ensure sufficient funding of the Health Platform. This points to the close ties between national and regional interests in the program.﻿ There are around 44,000 healthcare workers and 720,000 citizens in Central Norway. Of the three hospitals in the region, the university hospital in Trondheim, St Olav’s Hospital, is the largest. The university hospital will replace its current EHR from Cerner with Epic. The nursing homes and home-care services are supposed to replace the systems Profil and Gerica, and the GPs are supposed to replace their current EHR systems CGM (CompuGroup Medical) and System X. In addition, the Health Platform has identified 80 other information systems that will be replaced by Epic. According to both the Health Platform management and the GPs, the EHRs in the GP practices are generally quite good, and comparatively better than the EHRs in the hospitals, home-care services, and nursing homes. While most of the healthcare workers in Norway are public employees, the bulk of the GPs are self-employed. This means that each GP clinic in our study has a contract with its current EHR vendor.

The Epic system is an integrated suite of software that is supposed to cover the needs of the healthcare workers in the hospitals, nursing homes, home-care service, and GP clinics. It also enables patients to access their own healthcare data.

Preparing for the implementation of Epic requires thorough planning, often years, but the actual go-live period is only eight weeks, reflecting that the vendor has a very rigid and tightly run project plan for this period (Ellingsen and Hertzum 2019). After Epic is introduced in the organization, any changes to the system are supposed to be done by configuration. If a customer wants new development, Epic strongly encourages that this be done in collaboration with other customers (often across countries) through a common requirements specification. For example, recently there has been a collaboration between Epic customers in Switzerland and Denmark about the development of functionality for medication management (ibid.).

Epic was originally developed for the US market but is now being implemented in several European countries and most recently in the Nordic countries Denmark, Finland, and Norway. The actual implementation of Epic in Central Norway was initially planned to start in 2021, but it has been delayed by the Covid-19 pandemic and will not occur until 2022.

When it comes to implementation, the 64 municipalities in the region have an option to participate, i.e. it is not mandatory. This also includes the GPs who need to be convinced that participation will be beneficial for them:

‘I find that the GPs are positively expectant, but they are ... well, how should I put it ... skeptical by nature and need a good assurance that this will be good. I tell them: we cannot force you; we will have to tempt you. This solution must be better than what you have today, and the economy must not be worse.’ (Director, medical GPs).

Consequently, it is not surprising that from early on, extensive user participation was identified as crucial to the success of the program, both for creating ownership to Epic and for ensuring the appropriate functionality (Ellingsen and Hertzum 2019). The Health Platform has involved the medical association nationally, regionally, and locally in the program. The association has an observer role on the program board, and they are part of the health professional reference group. During the progression of the program, the Health Platform has consulted 400 clinicians from the entire healthcare service in Central Norway. Overall, the program has organized 101 workshops where users specified what they would need from Epic. The outcome of these workshops became the basis for the requirements specification, which included 4000 requirements. As a large-scale suite system, it is expected that most of the user requirements can be accommodated by the configuration possibilities in Epic - before, during, and after implementation - instead of development. In particular, the GPs will be invited to contribute in configuring the workflows and content of their solution. A key reason for this invitation is that the GPs have a particularly important role in the coordination of the municipal health services. Their participation is therefore crucial to the success of the program.

However, despite the thorough preparations, the Health Platform management expresses some uneasiness about getting the GPs on board:

‘Although we see that the GPs are positive and interested, we don’t know how many GPs we get recruited in the end.’ (Director, medical GPs)

It has been a source of concern that several studies and media have reported troubles with Epic. For example, the implementation of Epic in Denmark in 2016 caused trouble, including adverse events, wrongfully sent referrals, integration issues, and delays (Hertzum and Ellingsen 2019). Two years after go-live, it remained uncertain to what extent productivity was still lagging behind baseline (ibid.). There has also been issues around translating the US-based Epic to a Scandinavian context. This relates particularly to how it standardizes the roles for nurses and physicians, resulting in less flexibility for collaboration, such as access to each other’s documentation. Moreover, the US reimbursement regime necessitates time-consuming documentation (Allen 2019). These issues are not lost to the Norwegian GPs. One of the directors explained:

‘Members of the medical association have participated in reference visits to Denmark to see Epic in real life, and there they got the impression that this might not have been so successful, but they have expressed that they want to contribute to success here in Norway.’ (Director, municipalities)

Also, American clinicians have raised concerns about the effect Epic has on local practice. In a chronicle in the New Yorker, Gawande (2018) reflects on how initial promises of efficiency with Epic resulted in the complete opposite. Similarly, Schulte and Fortune (2019) analyze how doctors now spend half their day or more engaging with Epic, clicking pulldown menus and typing rather than interacting with patients, leading to doctor burnout.

## Findings

In this section, we present the content of the four themes that relate to what the Health Platform management and the GPs expect of the implementation of Epic in terms of organizational effects. For each of the themes, we contrast the views of the management and the GPs in order provide a broad understanding of the theme as well as point to potential challenges for the future implementation. The themes are as follows:

- Accessing patient information anytime, anywhere.
- Standardizing patient pathways.
- An EHR that responds to evolving needs
- Relocating work.

### Accessing patient information anytime, anywhere

In alignment with the long-term national goal of establishing a nationwide EHR functionality, “one citizen - one record”, the goal in the Health Platform program is to create a comprehensive health service that is well connected within the hospitals and across the hospitals, nursing homes, home-care services, and GP clinics. The aim is to provide all health personnel – and patients – in Central Norway with complete and up-to-date information about the patients’ condition and medication. The management explains that eighty existing systems will be replaced by Epic, which is particularly welcomed in the municipal health service (nursing homes and home-care services) where modernization of the EHRs is much needed. The regional scope of Epic also means that there is little need for integration among systems because all the patient’s data are expected to reside in Epic. This will improve data quality:

‘It is important that health workers understand that this means an improvement also on the integration side. Because today they actually print documents from different systems and enter this data into Doculive [the current EHR] with the sources of error this can cause. So, hunting for duplication of this kind of work for especially the medical group, is something we are going to do intensely in the preparation phase.’ (Director, top)

Management also expects much improvement in access to clinical data for healthcare professionals, including GPs:

‘When the EHR is updated, everybody [with proper access rights] can see the data. In a way, it will be the kind of core electronic record we have been dreaming of since 2004. Also, the patients can log on and look at their data.’ (Director, municipalities)

‘The biggest change for GPs is that they do not have to look in a continuous patient record: "When was the last time you were here: one year ago ... oh, well ... was it two years ago?" They will have a database that provides everything they need in real-time and just in one workspace with good overview’ (Director, municipalities)

However, some of the GPs raised the concern that the mere possibility to access the hospital record to find additional information about their patients might create expectations about looking it up just to be sure. One of them stated:

‘The problem is that when we have the whole hospital EHR at our disposal, it becomes an active choice not to use it.’ (GP-4)

To illustrate the point, the GP compared the situation with how physicians generally want not to over-diagnose patients:

‘If we can compare the situation to diagnostics. Physicians try not to order each and every test and image examination and everything like that because if they find something, they must follow up on it etc. This does not lead to better health; it instead becomes a kind of precautionary medicine.’ (GP-4)

The GPs’ views reflect a concern about the usefulness of Epic in their daily work in their GP clinics. The overall worry for the GPs is to be burdened with too much information. They have no wish to access the whole hospital record to read all the details of what has happened with a patient during a hospital stay. Several of the GPs state that they almost drown in information already and that having to read even more would be an additional burden.

‘A great amount of data in the hospital record is not relevant for us.’ (GP-3)

What the GPs consider essential, though, is to receive information that is relevant for their follow-up and treatment of a patient, most prominently the discharge reports from the hospitals. The GPs explain their use:

‘What matters to a GP is the reason the patient was admitted, what assessments were made during the hospitalization, and what should be followed up (...) For example, in a way, it is good that the surgeons write short discharge reports because the technicalities around a surgery are not that useful for us.’ (GP-3)

Generally, the GPs are quite happy with the discharge reports they receive from the hospital. If the GPs need additional information, they prefer to talk to the hospital physician who wrote the report rather than to try to find the sought-for information in the hospital EHR, “because in that case, the report is missing something” (GP-9).

For their part, the Health Platform management invites the GPs to see the potential in a shared system, i.e. a system that is useful to many user groups. It also tries to appeal to the GPs by arguing that there might be some benefits for them.

‘We tell them that some things will become easier: Today, the GPs don't have information in real-time, they are dependent on an electronic discharge letter. If the GP meets a patient who was at the outpatient clinic yesterday, they may not get the report until a week later, and that is a drawback.’ (Director, medical GPs).

To some degree, the GPs acknowledge that access to information that presently is unavailable to them might be useful. According to one of the GPs, an example might be when hospital physicians request tests on a hospitalized patient without putting the GP as a copy requisitioner. Then the GP will not see the result.

### Standardizing patient pathways

An essential goal in the Health Platform program is to streamline workflows and standardize patient pathways across organizational boundaries. Standardization is expected to ensure smooth workflows where each step is predefined and necessary information is available. For each workflow step, Epic should require the prescription of certain medications, the administration of certain tests, and the order in which the tests should be administered. In the outpatient clinic, standardization is for example envisaged for referral letters from the GPs. Here, Epic will be able to impose standardized content:

‘For example, when a GP refers a patient to us for a hip transplant examination, we want to standardize what information is necessary: we require certain results from the GP, we require a certain x-ray examination, we require certain blood tests, and we require a certain function score.’ (Director, medical hospitals).

In addition, standardized patient pathways are closely connected to structured content of the major documents. Structured content will serve as the foundation for warnings whenever a registration is outside defined limits. It may also serve as the foundation of decision support in both this step and later ones. Furthermore, structured content is expected to provide for more efficient reporting and research. Predefining the order of tasks for patients with certain conditions could make patient visits to hospital more efficient. It may ensure that the patient can go through required examinations in one visit instead of multiple visits. Still, such standardization is expected to make the work process more rigid:

‘This will be new to everyone: You need to enter some data before you can move on to the next step in the workflow. Therefore, we have to make sure there will not be too many hard stops. And we will ensure that you can get help, if needed.’ (Director, medical GPs)

The Health Platform managers recognize that it takes longer to work in a process-oriented system such as Epic, both to fill in the required data and comply with the structured form. However, the managers believe that the contribution of a shared EHR to the strategic goal of “one citizen - one record” outweighs the costs for some user groups:

‘Getting the users to embrace the solution and getting them to see the big picture really improve the overall quality of the healthcare service. It may mean more clicks for you, but you perform these clicks for your patient and for your colleagues.’ (Director, top)

After establishing a standardized patient pathway for one municipality, the Health Platform aims to disseminate it to other municipalities to ensure efficient work processes across the health region. This is supposed to ensure that all patients get the same level of treatment and care. The standardization of work processes across healthcare contexts is also attractive in a maintenance perspective because it will ensure that those who configure these processes in Epic, will only need to maintain one work process.

The GPs for their part are aware that the process-oriented nature of Epic will be a huge change to their work practices. They will have to start documenting in real-time where their tasks are but one step in a larger, standardized, and structured work process. They are troubled about this and concerned about the local consequences:

‘There is a lot of focus on making new patient pathways, but it will require a lot of work to do the necessary adjustments... I think it will be difficult to configure a platform that fits all the medical offices and the nursing homes. I really don't think it's possible.’ (GP-7).

The GPs are also worried about how Epic inscribes a fixed pattern of use:

‘What worries me as a GP is the rigidity of the system with structured documents (…) In order to move forward in the registration process, you must have a structured [entry], but I don’t have time to struggle half an hour for producing a referral letter because it lacks some code.’ (GP-8)

The GPs are not unfamiliar with structured data entry. Today, some blood-pressure values are archived as numbers to make them easy to extract, compare, and present in a laboratory sheet instead of reading the values from the notes. While this is considered useful, the GPs simultaneously point out that structured data entry is a lot of work for the user:

‘A lot more is required from the user if there are lots of boxes to click or selections from a list. Instead, we use many abbreviations for Latin words that are demanding to write. So, it often becomes "A." or something for artery, instead of writing it out, looking it up, or finding it.’ (GP-1)

To underscore the point of extra work related to structured content, the GP referred to a case where they had experimented with going from a paper-based medication chart to a structured electronic medication chart:

‘After requesting 1 liter of Ringer, i.e. liquid, you could write *"Ringer, 1000 ml i.v., x 4*" on the paper-based medication chart. In comparison, on the electronic chart there were 18 clicks, because first you had to choose fluid treatment, intravenous, find which fluid, what volume... Yes, it was a lot of things. That's the danger of structuring.’ (GP-1)

### An EHR that responds to evolving needs

In the preparation phase, Epic will be configured for many different settings: hospital departments, nursing homes, home-care services, and GP clinics. Given Epic’s large scale, a regional organization under the direction of the Central Norway health authority will have the responsibility of running the system. This organization will also have the responsibility for coordinating configuration activities after the system is put into operational use, thus responding to evolving user demands. The management considers this a big advantage and compares it will traditional EHR development:

‘This is a completely different division of labor between the customer and the supplier than we have been used to. Until now, we have had to specify requirements and deliver these to a supplier who has then developed the system. This will happen to a much lesser extent here because Epic is such a configurable system.’ (Director, top)

‘You do not have to do what you have traditionally done - to call a supplier who does not have time to do this or that. It takes an eternity before something can be done. Here it is so configurable that you can set up, fix and mix (...) then you run what Epic refers to as continuous optimization processes that you as a customer control yourself. You are actually building this system yourself.’ (Director, benefits realization)

The Health Platform management has presented two benefits for the GPs. It has been arguing that the GPs will be released of the cumbersome responsibility of running an EHR solution - a responsibility that every autonomous GP clinic has today. The GPs will also be released of the responsibility of coordinating software-change initiatives vis-à-vis a vendor because such changes now can be configured.

Despite this, the core of Epic is still a large-scale system with common functionality. There are limits to what it can be configured to do, both in response to the GPs’ technical requirements and in relation to standardized patient pathways (see Section 5.2). The Health Platform management sees this as a natural consequence of implementing a shared system for the whole region:

‘I think some users will lose some of the most specific functionality of their specialist system, but I think they are willing to sacrifice this when they see the benefits of sharing information with others.’ (Director, top)

However, the point for the GPs is not only about losing some functionality in their daily practices. It also involves strategic considerations about what type of system they would like to have for the future. The GPs are skeptical toward Epic because it represents a “closed” large-scale suite system. One of the GPs questioned whether it made sense to invest in such a closed platform and felt that discarding more open systems was like going back in time. Another raised a similar worry:

‘I fear that Epic will be a huge colossus... It may not be as user-friendly as one had hoped for.’ (GP-8)

Several of the GPs emphasized that it might be better to have several small and lightweight EHRs that are capable of communicating with other parts of the healthcare sector through message-based services. An example of such EHRs is the GPs’ present EHRs. These EHRs stand in contrast to Epic, which is perceived as big and heavy.

The GPs find that their EHRs are intuitive and user-friendly. Typically, new employees and interns do not need much training before they can use these systems. The GPs are also happy with the support from their vendors. They receive support over the phone, and if needed the vendors can access the EHR remotely and fix problems. For the CGM system, the users and the vendor share a Facebook group where they frequently interact and exchange advice. Several of the GPs find it very useful. One of them put it like this:

‘We support one another indirectly because if someone asks a question that someone else has answered earlier, we often respond in pure solidarity: "You do so and so...". This takes ten seconds and then they are online.’ (GP-6)

Ideas for new EHR functionality may also be presented and discussed in the Facebook group, and changes may happen quite quickly. One of the GPs contrasts this situation with how their hospital colleagues experience the vendor relationship:

‘When the physicians at the hospital call [the vendor] with an IT question they may not get anything solved in six months. In comparison, I experience that our vendors are quite good at solving things.’ (GP-9)

The GPs experience almost no system downtime with their current EHRs and are, therefore, concerned over reports from Denmark about downtime with Epic. As potential users of Epic, the GPs worry that they will not be first in line for getting help in case of system downtime:

‘If the system crashes, I suspect that the hospital will be prioritized over us. And that would be dramatic for us. As self-employed GPs, we have no income when the system is down.’ (GP-4)

Another concern for the GPs is how Epic may be used for managerial purposes. Given Epic’s region-wide scope, they are worried that it may be used for surveillance and control, for instance to monitor the number of patients per GP, the time spent on each patient, etc. According to one of the GPs, it is extremely important that the integrity of the GP clinics is maintained and that these clinics control the access rights of “outsiders” who want access.

In sum, the GPs are not resisting a system change per se. Actually, they realize that a system change will be needed in the not so distant future to stay abreast with the technological development. However, they regard other alternatives as more promising than the “heavy” Epic. Many GPs would prefer to pursue more open and innovative systems that follow a technological pattern (small and light) similar to their present systems.

### Relocating work

The strategic concerns of Epic include a long-term dimension in which the implementation of Epic will make it possible to move tasks across sector boundaries.

‘Depending on how the [Norwegian] financing system eventually turns out, there will be some relocation of tasks. But it is quite clear, all the health trusts have their development plans where 30% of what they do at the outpatient clinic today will go to the municipalities.’ (Director, medical GPs)

Along similar lines, one of the GPs explained that the university hospital in Central Norway plans to transfer 10–15% of its tasks to the GPs (GP-3). This will be possible because all health professionals use the same system and have access to the same information in real time. Many find this possibility to be an essential motivation for participating in the implementation of Epic:

‘If this program had only been aiming for the hospital sector, it would have been really demotivating (…) But since the aim is to procure a system for the whole healthcare service in Central Norway with its future potential, it warrants taking a risk like this.’ (Director, medical hospitals)

Nonetheless, for the municipal health personnel, the relocation of work includes more responsibility in relation to the monitoring, treatment, and medication of the patients in nursing homes and home-care services. In this regard, the Health Platform management considers it crucial to efficient coordination that everybody use the same system:

‘[When it comes to really ill persons in the municipalities], it is extremely important that the nursing staff in the municipalities, the specialists in the hospital, and the GPs are on the same system, and especially the GPs who are the ones who will do most of the coordination work.’ (Director, medical hospitals)

However, the GPs are worried about the prospects of increased coordination responsibilities. One of them put it like this:

‘Obviously, we are much better at detecting cancer in our patients than they are in the hospital because we know them (...) But I can't follow up on a cancer coli Dukes B or whatever it is called without knowing what it is, without having time to learn it, and without getting paid for it. So, it must be a second step. I'm afraid that having all these opportunities with the Health Platform will drag us down completely.’ (GP-2)

While these worries concern the future, the GPs see the implementation of Epic as an immediate driver for many new tasks and responsibilities that are coming their way. They are therefore protective of their current work situation:

‘I receive around 40 discharge letters daily and maybe 100-150 lab results. I process a to-do list after seeing the last patient, I have the e-prescriptions and a pile of documents. We don’t want this pile of documents to grow any larger. So, we are very concerned about how we should shield ourselves from new tasks when Epic comes about.’ (GP-3)

An illustration of a new task introduced in the health service in Central Norway is the dialogue-message service that was set up during the autumn of 2018 (i.e. prior to Epic). This service allows for asking short free-text questions about a patient’s treatment and care. It enables the staff in the nursing homes and home-care service to ask questions to GPs (and vice versa). However, the GPs experience that the messages from the municipal health service come straight to the GPs without any screening. One of them elaborated on a typical message: What diagnoses did this patient have when he was with you last time, what are his regular medications, what happened today when he was with you? From the GPs’ point of view, the home-care staff uses the message service as a chat function where they ask quite trivial questions and expect an answer straight away. While the Health Platform management has said to the GPs that this message service will be discontinued after Epic is implemented, this is an illustration of but one new task that the GPs have to manage. They are concerned about the present trend of getting a lot of unscheduled tasks. In this regard, the Health Platform management has suggested that if the GPs participate in configuring Epic, they will be able to exercise more influence on the interorganizational workflow and will take part in deciding how work is distributed.

Still, the GPs are not convinced. As they see it, some of the potential changes to their work situation will happen because Epic provides opportunities that are seized by some staff, but unattractive to the GPs. One GP provided an example related to accessing their appointment books:

‘It is evident that it would be very beneficial for the nurses and the physicians at the hospital to be able to say: “GP NN has a free slot in 14 days, you [the patient] can have that one for the in-between controls.’ (GP-2)

While this example is about what might happen, the GP substantiates the concern with a recent experience with a hospital specialist:

‘An orthopedist wrote to me: "Request that the GP orders an MRI of the spine before the next outpatient check”, and then I had to answer: “Thank you very much for having confidence in me, but I believe you can manage this yourself. Sincerely, NN”. We cannot have it like that.’ (GP-2)

The GPs are concerned that they will increasingly get a role as secretaries for the hospital physicians.

## Discussion

A main characteristic of an information infrastructure is the installed base, that is, the existing systems, users, and practices (Bowker and Star 1999; Aanestad et al. 2017; Aanestad and Jensen 2011). Central Norway health region is an illustration of the existing installed base, both related to technologies and existing practices. The socio-technical ensemble in Central Norway has not been built from scratch; it always builds on and extends the existing one (Aanestad et al. 2017). Thus, new technological components are added in an incremental manner, which in turn may gradually attract new users (Hanseth and Lyytinen 2010).

The implementation plan for Epic challenges this incremental view. In accordance with its business model, Epic will replace the currently used systems in one single stroke and at the same time keep the users in-line. Among information infrastructure scholars, “[a]pproaches that neglect to deal with the installed base are often called ‘installed base hostile’” (Aanestad and Jensen 2011, p. 162–163). This hostility makes it harder to deal with evolving issues, side-effects and users’ fluctuating interests, which all are key to the information infrastructure concept. We move on to discuss this theme along two dimensions in the following subsections.

### Generic systems and the forces of standardization

In our literature review, we have pointed out that implementing information infrastructures involves both national and local concerns, and they are frequently in a tensional relationship (Star and Ruhleder 1996). Tensions can also be found in our case where the Health Platform management promotes the regional goals of the program, while the GPs have local concerns. However, we expand on this perspective, by elaborating how generic systems shape the relationship between the regional and the local. We also point to how the GPs as independent entrepreneurs enjoy considerable negotiation power in the decision process. This prepares the ground for a situation where various stakeholders need to negotiate and compromise to secure progress and success in the project (Latour 1987).

A generic system represents in itself a powerful actor (Latour 1987) given its size, scope, and complexity. It not only enforces the tension between regional and local concerns, it eables a shift in the power balance in favor of the regional perspective. In our case, the regional goals are very ambitious as they are connected to the ambition of realizing the national goal “one citizen - one record”. The means to achieve this goal is Epic, through its powerful standardization capabilities: First, despite all its configuration possibilities, Epic is still a system that promises the seamless integration and standardization of information and work processes within and across organizational areas (Kumar and van Hillegersberg 2000, p. 23). Second, Epic’s capability for structuring its content is related to external and internal reporting, integration of data across sectors, foundation for decision support and research, and input to graphs and trends etc., for instance related to the patient’s medication list. Third, the establishment of standardized patient pathways defines what kind of work tasks should be performed and in which order. As we have pointed out, for a given patient condition Epic can require the prescription of certain medications, the tests to be taken, and the order in which to take them. This type of standardization initiatives make a powerful and appealing tool for health authorities and decision makers who aim to streamline organizational processes (Pollock et al. 2003; Saxena and McDonagh 2018). Moreover, in a long-term perspective these various forms of standardization prepare the ground for easy relocation of work, i.e. deciding who should do certain work tasks.

In sum, the forces that drive standardization are immensely powerful and well aligned with the regional interests. It is therefore not surprising that the Health Platform management argues that the GPs must be prepared to lose some of the most specific functionality of their current systems, i.e. an encouragement to see the bigger picture and not just think of their own self-interest. This suggests a strong pull toward regional and collective priorities at the expense of local ones. Unfortunately, this view may prove to be an added risk factor. Aanestad and Jensen (2011) emphasize how hard it is for large-scale projects that are geared towards future, collective benefits to achieve wide enough stakeholder mobilization (p. 173). The alternative would be to offer specific benefits to the GPs to encourage and facilitate their participation.

Additionally, former studies have pointed to how large-scale projects in healthcare easily run into problems, produce unintended consequences, or ultimately fail (Sholler 2020; Pollock and Williams 2010; Hertzum and Ellingsen 2019). Even one of the directors in our case emphasizes that the Health Platform is a high-risk project. It appears fair to conclude that these projects are unpredictable in terms of progress, outcome, and organizational consequences. What shouldn’t have been so hard to predict though, is that the GPs would hesitate to adopt Epic. The GPs are concerned about the degree of standardization, the amount of information, and the prospects of additional work. At the same time, the GPs are fully aware that the Health Platform is dependent on their participation and cooperation for realizing the full potential of Epic as underscored by one of the directors:

‘While it is important for us in the specialist health service that the municipalities join, it is even more important that the GPs join because in medical terms our partner is the GP, and we who work with symptoms and diagnostics on a large scale must be on the same team.’ (Director, medical)

The participation of the GPs is not only crucial for the specialist health service, but also for the municipalities because the GPs are coordinating their health services. For this reason, if the GPs do not opt in, the municipalities will probably not opt in either. This puts the GPs in a gatekeeper position (Latour 1987) that gives them some influence. The GPs have now moved forward and strengthend their united negotiation power by mobilizing the medical association to be their representative in the coming negotiations with the Health Platform program.

The prospects of negotiations and compromises – in which both sides have to give and take – stand in contrast to the rather rigid setup of the Epic project. This rigidity requires that the future users adhere to Epic’s playbook. For the GPs, we can conclude that they will not play along unless some key conditions are met. As a result, the inevitable negotiation process will add to the existing uncertainty about the implementation of Epic and its outcome. In addition, based on these negotiations, the current pull towards regional interests (driven by the Health Platform program) may change towards a stronger local pull (driven by the GPs) when the consequences of the system become clearer (Cresswell et al. 2020).

### Small-scale versus large-scale systems

Part of the existing installed base in Central Norway, is the GPs’ existing EHRs. These systems are relatively small and self-contained, and they are controlled by the individual GP clinic. Presently, these systems are well-integrated with the other systems in the healthcare sector: with the hospitals (referral letters and discharge reports), with the home-care services and nursing homes (dialogue-message services), and with national services (the national core record, citizens’ access to their health information, and e-prescriptions). There are several vendors in the Norwegian market for these GP systems, and if needed, the GP clinics can replace their EHR with a system from another vendor. An example is how several clinics in Central Norway currently are replacing System X with PatientSky due to a recent acquisition in the market.

These GP systems reflect a rather loosely coupled information infrastructure (Hanseth and Lyytinen 2010). Evolving the infrastructure can be done through changing parts of it (systems or people) in a stepwise and modular manner. This type of modularity consists of relatively self-contained systems with standardized interfaces (Aanestad and Jensen, p. 172). In contrast, modularity in generic systems consists of functionally integrated domain modules within the overall solution. This means that the evolution of generic systems is primarily based on the continual configuration of the system.

While the Health Platform management signals that the GPs are cautiously optimistic about Epic, our data suggest that they are worried. As independent entrepreneurs, the GPs get paid per consultation, and any unexpected incident that might delay their activities during the workday will have a direct impact on their paycheck. Therefore, the GPs consider what kind of EHR system can support their entrepreneurial role. Our data show that the GPs’ current systems invite local and improvised approaches (Orlikowski and Hofman 1997) for changing the software. The GPs explain that they can suggest, discuss, and act upon new ideas in an informal way through Facebook groups, phone, or email. Hence, the GPs have ownership of their systems and can exercise some control over how they should evolve. In this regard, the GPs find that Epic is a “huge colossus” that may work against their interests in the long run. They would rather have small and lightweight systems on which they can have more influence. This influence would, for example, enable them to tailor patient information to their needs as well as ensure that they get support when the system is down or inexplicable things happen.

Another important issue relates to being able to clarify the boundaries between the GP’s domain and other professional domains. First and foremost, the GPs don’t want access to all the information available in the EHR. They are quite happy with the message-based approach that is based on referral letters to hospitals and discharge reports from hospitals. Second, the GPs also want to limit other professionals’ access to their domain. For instance, they don’t want the care staff in nursing homes and home-care service to be able to access their EHR too easily because that will drain their resources. Similarly, they don’t want the specialists at the hospital to exercise control over functionalities such as appointment books in the GP practices. While the Health Platform management has promised the GPs an influential role in preparing for the Epic implementation, the above-mentioned concerns are perhaps something they are not willing to give because the whole point with Epic is to reduce boundaries between domains, not to sustain them.

In comparison to small-scale systems that rely on integration, large-scale generic systems rely on their configurability. Epic (or the management) claims that it can be configured for the local practices and users in Central Norway. Users are then invited to take part in tailoring user interfaces, adding content, and setting up workflows and information flows. In addition, a hierarchy of formal decision fora has been set up to devise a negotiated solution that is acceptable for all (Hertzum and Ellingsen 2019). In this way, configuration is a means to get the GPs on board, make something useful for them, and ultimately motivate them to accept the system.

Simultaneously, the Health Platform management must carefully consider the regional goals related to Epic, i.e., structuring the EHR content, real-time registration, and standardization of work practices. In pursuing these goals, the configuration activities should prioritize regional concerns that span multiple organizational domains and levels (Saxena and McDonagh 2018). This way, configuration becomes a means for standardization (Pollock et al. 2003) on a wide-ranging scale where the GPs are but one voice among many others. Due to the broad scope of the processes and the many users involved, local GP requirements may have to yield to “the common good” as emphasized by one of the managers. By shifting design from individual GP needs to region-wide needs, the vendor and the Health Platform management move the configuration of Epic from the domain of each user site, where only particular needs can be articulated, to a public setting, where more general requirements can be forged (Pollock et al. 2007, p. 263). Just consider how the Health Platform invites the GPs to participate in configuring Epic by promising them more influence on the inter-organizational workflow and how work is distributed. Accordingly, it may be difficult for the GPs not to participate. It is only by participating that their needs can be effectively represented (Pollock et al. 2007, p. 267).

A related matter is that a lot of the configuration occurs in the preparation phase, before Epic goes live. While thorough long-term planning and preparations are comforting per se, they point to a problematic consequence for the GPs’ possibilities to get new functionality in the long run. The long-term planning needed for configuring Epic for regional purposes, along with the involvement of all the implicated users, constitutes a substantial initial effort (Ellingsen and Hertzum 2020). This may result in a system that is so standardized when it “goes live” that it will be hard to change at later stages. If so, emergent opportunities and unanticipated consequences will remain unaddressed because the users are discouraged and prohibited from modifying the system during use (Germonprez et al. 2011). In this regard, the Health Platform fulfills Bowker and Star’s (1999, p. 135) point that “[s]oftware is frozen organizational and policy discourse”.

Moreover, configuration is limited to what technically is on offer from Epic, which seeks to maintain the system’s generic characteristics (Ellingsen and Hertzum 2020). Generally, it is therefore not possible for customers (and even less for users) to request the development of new functionality unless several customers ask for it and it is developed as a generic capability (Pollock et al. 2007). In this regard, the GPs in our case will lose the possibility they currently have to communicate ideas for new functionality directly to their vendors. Instead, adaptations will basically be restricted to what is possible within a configuration regime.

The business plan states that after the implementation period the vendor will pull out and leave the further responsibility and configuration activities to a Norwegian regional organization that is supposed to evolve the Epic portfolio in the future. This strategy may prove problematic: A strategy that is solely based on configuration and, in principle, does not include any development makes it difficult to integrate Epic in the regional installed base and to incorporate new innovative components in Epic. This is neither supported through Epic’s technology nor through its policy.

More generally, the Health Platform program has actualized – and polarized - the national discourse of strategically choosing between large-scale and small-scale systems. On the policy level, there has been a clear tendency to privilege large-scale initiatives under the umbrella term “one citizen - one record” because these carry the “promise” of efficiency, sweeping changes, and access to patient information whenever needed. However, these initiatives have met increasing resistance among healthcare users, ICT vendors, and researchers who have argued that this is a risky and old-fashioned strategy. Recently, the Norwegian Auditor General (2021) conducted an evaluation of the Government’s strategic health ICT initiatives. It concluded that that the authorities lacked control over these initiatives; they were too big, too complicated; and they failed before they had even started. Therefore, many argue for an alternative that allows for the development of an eco-system where different modular systems interact with one another through standardized interfaces. It is argued that such an eco-system will help ensure that various user groups get functionality tailored to their specific needs, will lower costs, and will increase industrial competition and innovation possibilities (Ellingsen et al. 2021). A telling illustration is how the Stockholm healthcare region in Sweden has recently decided to acquire a module-based portfolio with many vendors at the expense of Epic.

## Conclusion

In this article, we have investigated the prospects for generic systems from the perspectives of health program managers and GPs. Already in the preparation phase prior to go-live, several promises and expectations are put to the test, which will influence the later stages of the program. This makes insights into this early phase important.

To ensure success with a generic system, it is necessary that the targeted user groups participate. The key reason for this necessity is that generic systems come with inherent characteristics of standardization that cut across the involved practices. If a key user group such as the GPs opts out, the touted end-to-end seamless workflow will suffer, and the generic system will probably then be a system for the hospitals. As a result, it is difficult to implement generic systems in heterogeneous healthcare practices with a distributed governance structure and very different professionals. The Health Platform program must handle the implementation carefully.

A dilemma in recruiting GPs for participation is that management must promise the GPs some benefits and, at the same time, promote the argument that the GPs must sacrifice some of their needs in the interest of the “common good”. For the moment, the benefits are not obvious. A way forward may be to test the overall setup by including a few willing GP clinics to see what the solution may look like in real life, and then experiment with a configuration acceptable to both parties. However, this requires that the Health Platform and Epic change their big-bang implementation strategy. This might not be easy given that Epic’s implementation plan follows a set course that is independent of the context in question.

We have discussed what role configuration plays in this process. Configuration easily results in the standardization of work practices, hence privileging national and regional concerns at the expense of the GPs’ concerns. In turn, this standardization may constrain local initiatives and innovations. A possible way forward is to postpone some configuration activities until the system is up and running. After all, a promise with generic systems is that they can be configured continuously after they have entered into operational use. By postponing configuration activities, it might be easier for the users to see the consequences of different configuration and standardization decisions.

As researchers, we have followed the Health Platform program since 2018, and we plan to continue doing this in the coming years. The implementation is scheduled to occur in 2022 and may not be a smooth process. It will be interesting to see how the process unfolds and how different stakeholder groups shape the outcome. Given the program’s increasingly high public profile characterized by many diverging “voices”, we believe it is an opportunity for CSCW researchers to contribute with nuanced accounts of what is going on. Such accounts can provide a basis for informed decision-making in - and around - the Health Platform program.
